# Attrition in HIV care following HIV diagnosis: a comparison of the pre‐UTT and UTT eras in South Africa

**DOI:** 10.1002/jia2.25652

**Published:** 2021-02-18

**Authors:** Dorina Onoya, Cheryl Hendrickson, Tembeka Sineke, Mhairi Maskew, Lawrence Long, Jacob Bor, Matthew P. Fox

**Affiliations:** ^1^ Health Economics and Epidemiology Research Office Department of Internal Medicine School of Clinical Medicine Faculty of Health Sciences University of the Witwatersrand Johannesburg South Africa; ^2^ Department of Global Health Boston University School of Public Health Boston MA USA; ^3^ Department of Epidemiology Boston University School of Public Health Boston MA USA

**Keywords:** HIV, attrition from HIV care, ART adherence, Universal Test and Treat, same‐day initiation of ART, HIV, ART access, attrition from HIV care, ART adherence, Universal Test and Treat, same‐day initiation of ART, HIV, attrition from HIV care, ART adherence, Universal Test and Treat, same‐day initiation of ART, HIV, ART access, attrition from HIV care, ART adherence, Universal Test and Treat, same‐day initiation of ART

## Abstract

**Introduction:**

Policies for Universal Test & Treat (UTT) and same‐day initiation (SDI) of antiretroviral therapy (ART) were instituted in South Africa in September 2016 and 2017 respectively. However, there is limited evidence on whether these changes have improved patient retention after HIV diagnosis.

**Methods:**

We enrolled three cohorts of newly diagnosed HIV‐infected adults from two primary health clinics in Johannesburg from April to November 2015 (Pre‐UTT, N = 144), May‐September 2017 (UTT, N = 178) and October‐December 2017 (SDI, N = 88). A baseline survey was administered immediately after HIV diagnosis after which follow‐up using clinical records (paper charts, electronic health records and laboratory data) ensued for 12 months. The primary outcome was patient loss to follow‐up (being >90 days late for the last scheduled appointment) at 12 months post‐HIV diagnosis. We modelled attrition across HIV policy periods with Cox proportional hazard regression.

**Results:**

Overall, 410 of 580 screened HIV‐positive patients were enrolled. Overall, attrition at 12 months was 30% lower in the UTT guideline period (38.2%) compared to pre‐UTT (47.2%, aHR 0.7, 95% CI: 0.5 to 1.0). However, the total attrition was similar between the SDI (47.7%) and pre‐UTT cohorts (aHR 1.0, 95% CI: 0.7 to 1.5). Older age at HIV diagnosis (aHR 0.5 for ≥40 vs. 25 to 29 years, 95% CI: 0.3 to 0.8) and being in a non‐marital relationship (aHR 0.5 vs. being single, 95% CI: 0.3 to 0.8) protected against LTFU at 12 months, whereas LTFU rates increased with longer travel time to the diagnosing clinic (aHR 1.8 for ≥30 minutes vs. ≤15 minutes, 95% CI: 1.1 to 3.1). In analyses adjusted for the time‐varying ART initiation status, compared to the pre‐ART period of care, the hazard of on‐ART LTFU was 90% higher among participants diagnosed under the SDI policy compared to pre‐UTT (aHR 1.9, 95% CI: 1.1 to 2.9).

**Conclusions:**

Overall, nearly two‐fifths of HIV positive patients are likely to disengage from care by 12 months after HIV diagnosis under the new SDI policy. Furthermore, the increase in on‐ART patient attrition after the introduction of the SDI policy is cause for concern. Further research is needed to determine the best way for rapidly initiating patients on ART and also reducing long‐term attrition from care.

## 1 Introduction

South Africa has experienced a dramatic increase in the number of patients initiating antiretroviral therapy (ART) since the start of the national HIV treatment programme in 2004. In 2019, around 60% of the nearly eight million persons living with HIV in South Africa were receiving ART [1, 2]. However, attrition from HIV care continues to threaten the overall success of the South African HIV treatment programme and the realization of the Joint United Nations Program on HIV and AIDS' (UNAIDS) 90‐90‐90 targets [3, 4, 5, 6].

South Africa has gradually expanded ART eligibility in response to evidence of the potential impact of immediate ART initiation on both patient health and onward transmission [7]. Initially set at 200 cells/µL at the start of the public‐sector programme in 2004, the CD4 count threshold for treatment eligibility increased to the WHO‐recommended 350 cells/µL in 2010 and 500 cells/µL in January 2015 [8, 9, 10]. In September 2016, South Africa introduced WHO guidelines for universal test‐and‐treat (UTT) of all HIV‐infected individuals, regardless of CD4 count [11]. In October 2017, after clinical trials demonstrated that rapid ART initiation could reduce patient attrition and increase viral suppression [12, 13, 14], South Africa further recommended same‐day ART initiation (SDI) [12].

While SDI was an important step forward, concerns remain as to the feasibility of the SDI policy given limitations in the capacity of public‐sector care in South Africa. Furthermore, not all patients are necessarily committed to life‐long ART on the day of HIV diagnosis, which could lead to a shift in attrition from pre‐ART care to post‐ART initiation [7, 15, 16, 17, 18, 19, 20, 21, 22]. However, net changes in programmatic losses from care covering both pre‐ART and post‐initiation care have yet to be quantified outside of trials as few observational HIV cohorts in South Africa collect reliable pre‐ART data to allow for comparisons [23, 24]. There is, therefore, a need for pragmatic research to clarify the practical impact of the SDI policy both on clinic practices and patient outcomes at the time of HIV diagnosis. Such evidence is essential to inform the design of interventions to support the policy implementation processes and translate clinical trial results in complex health systems.

In this study, we aimed to determine whether the implementation of UTT and SDI policies in the South African public health sector is associated with reduced attrition from HIV care, 12 months after HIV diagnosis.

## 2 Methods

### 2.1 Study setting and design

We conducted a prospective cohort study at two peri‐urban primary healthcare clinics (PHCs) in Johannesburg, South Africa. PHCs in Johannesburg are mainly nurse‐run with the support of one medical doctor. PHCs are responsible for HIV testing, ART initiation and primary care‐level management and monitoring of HIV‐positive patients. The City of Johannesburg metropolitan municipality is the largest of five health districts in the Gauteng province of South Africa. Johannesburg comprises 108 PHCs subdivided into seven regions or sub‐districts (denoted A‐G) covering about 75% of the population (mainly uninsured) [2]. This study was conducted at two (of 13) conveniently selected public‐sector PHCs in the Johannesburg health sub‐district A.

**Figure 1:**
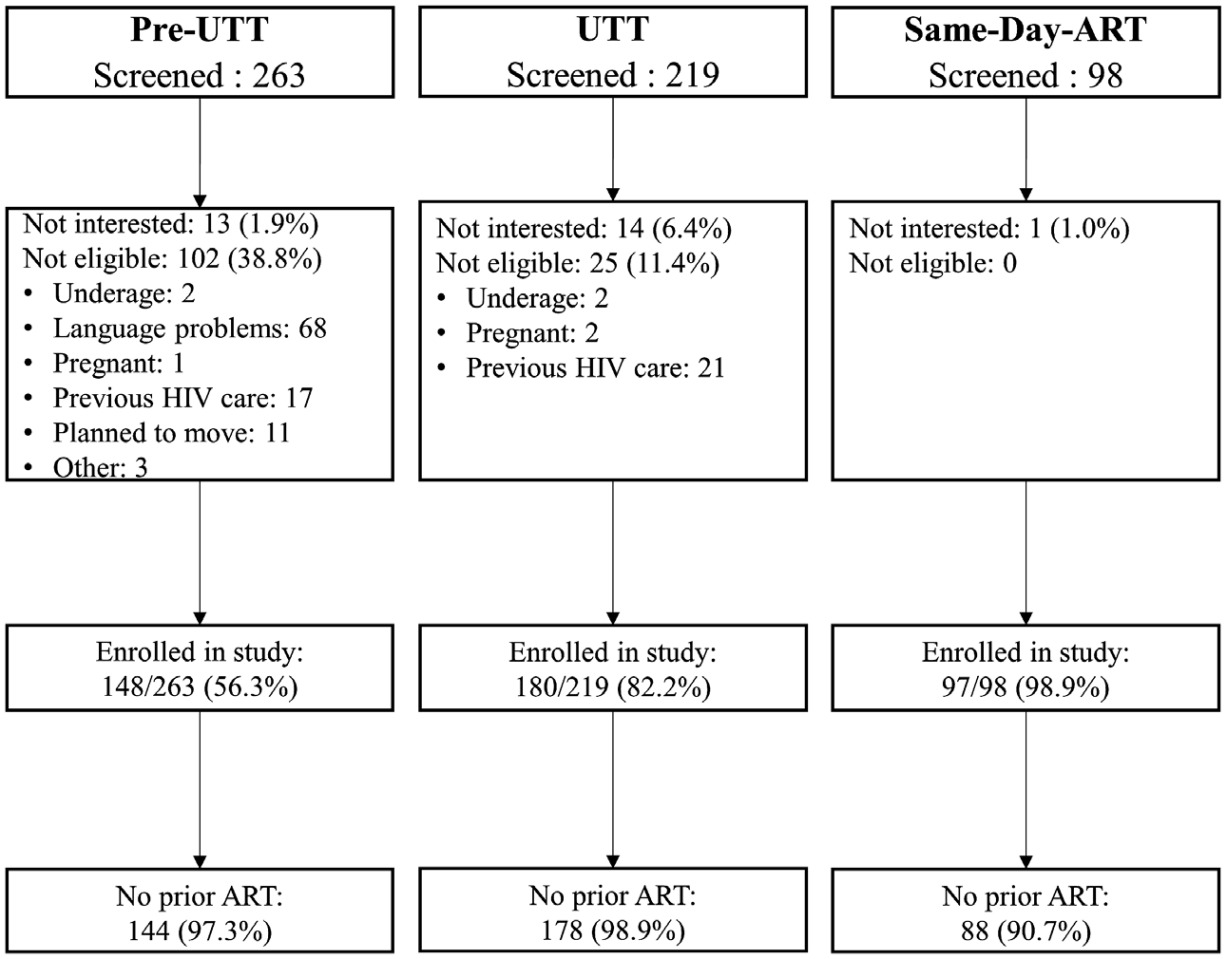
Participants flow across the pre‐UTT, UTT and SDI cohorts.

We enrolled newly diagnosed HIV‐positive adults (≥18 years) entering care based on the timing of ART policy changes: from April to December 2015 (CD4 < 500 or Pre‐UTT period), July‐August 2017 (UTT period, CD4 criteria eliminated) and October 2017‐August 2018 (SDI period, ART initiation delay eliminated) (Figure 1). All participants were enrolled in the study via referral from PHC‐based lay HIV counsellors immediately after an HIV‐positive diagnosis. All eligible and enrolled participants provided written informed consent.

HIV positive adults were eligible to participate if they self‐reported being newly diagnosed with HIV, had no prior history of ART and were well enough and willing to provide consent to participate in the study. Participation in the study included completing an interviewer‐administered questionnaire after the screening and consenting process, and giving permission for study staff to access medical records. Additional eligibility criteria included being conversant in either English, Zulu or Sotho and having entered HIV care after the HIV‐positive diagnosis. The consent process and interviews were conducted exclusively in English in the pre‐UTT cohort because we assumed that the urban Johannesburg population would be conversant in English but later translated to Sotho and Zulu as well for the UTT and SDI cohorts. Entering HIV care was defined as providing a first blood sample for baseline laboratory tests for the pre‐UTT and UTT cohorts. We defined entry in HIV care under the SDI policy as having received the HIV‐positive test result because new clinic processes meant that patients were likely to start ART before the first blood collection. Before UTT, the first blood tests were necessary to determine patients' CD4 count eligibility for ART and the appropriate initial ART regimen. Entry into care was assessed on the day of HIV diagnosis, and eligible patients were enrolled on the day of diagnosis as well. Women who were pregnant at HIV diagnosis were excluded from the study because ART initiation and monitoring processes in antenatal care differ from that of non‐pregnant populations. Study staff cooperated closely with lay HIV counsellors across sites and checked HIV testing records daily to ensure that all patients diagnosed with HIV were referred to study staff for study eligibility assessment.

### 2.2 Study sample size

Studies in South Africa have estimated that up to 70% of HIV‐positive patients with baseline CD4 counts 350 to 500 cells/µL fail to return within one year of their scheduled visit and that 60% of those with CD4 ≤ 350 cells/µL are lost in the same period [4, 5, 23]. The pre‐UTT (CD4 < 500 threshold) sample of 148, was calculated to detect a 20% differences in attrition between the exposure groups using an α of 0.05, 80% power. In calculating the combined UTT and SDI cohorts sample size, we further assumed that up to 70% of HIV‐positive patients with CD4 counts >500 cells/µL would fail to return within one year of their scheduled visit, and that only 25% of diagnosed patients would have a CD4 > 500 cells/µL. Given that the baseline CD4 could not be determined before enrolment and to ensure the enrolment of enough patients with baseline CD4 count >500 cells/µL, we oversampled by a factor of two. We enrolled an additional 276 HIV‐positive individuals after the UTT guideline (including 88 after SDI).

### 2.3 Data collection

participants completed an interviewer‐administered baseline questionnaire after HIV testing, on the day of diagnosis. Interview questions included demographic and socio‐economic characteristics as well as health‐seeking behaviour. The recency of the HIV diagnosis was self‐reported during the eligibility assessment. Participants whose medical records indicated prior ART were excluded from the analytic dataset. Patients were passively followed‐up by paper, and electronic (including laboratory data) routine medical record review from the date of HIV diagnosis until the last visit in the first 12 months of observation to determine attrition from care.

The study team extracted routine clinical data from the paper files and electronic HIV registers. Fields collected for the study follow‐up included HIV diagnosis date, baseline CD4 count, date of ART initiation and resource usage (visits, laboratory tests, ART collection). We defined baseline CD4 as the first CD4 result determined using blood specimens collected on the day of HIV diagnosis. All patient‐level data were collected using a study‐specific database designed and managed in REDCap (Vanderbilt University, Nashville, Tenessee) [25]. All datasets were zanonymized and exported to STATA 14 (StataCorp, College Station, Texas) for cleaning and analysis.

### 2.4 Outcome data

The primary exposure variable was the HIV treatment guideline at the time of HIV diagnosis: pre‐UTT (policy active between January 2015 and August 2016); the general UTT policy (policy active between September 2016 and September 2017) and the UTT/SDI policy (active from the end of September 2017) [8, 9, 10, 11, 12]. The primary outcome was being lost to follow‐up (LTFU), defined as being ≥90‐days late for the last scheduled appointment in the 12 months following HIV diagnosis [26]. Person‐time accrued from the date of HIV diagnosis (study enrolment) until the date of the last recorded clinic visit in the 12‐month observation period.

### 2.5 Statistical analysis

We calculated frequencies and proportions for categorical data and presented these results by ART guideline period (pre‐UTT, UTT, SDI). For continuous variables, we calculated medians and interquartile ranges (IQRs) due to the non‐normal distribution of the data. Following STROBE guidelines for observational studies [27], we present baseline descriptive results without statistical testing. Kaplan–Meier analyses were conducted to assess time to becoming LTFU in the first 12 months of HIV care. We compared the proportion considered LTFU at 12 months between treatment guideline periods using crude and adjusted Cox regression modelling reporting hazard ratios (HR) with corresponding 95% confidence intervals (95% CI). Predictors of variables with a *p* < 0.1 in crude analyses were entered in the multivariate model. Schoenfeld residuals were used to test the assumption of proportional hazards. ART initiation has been found to influence attrition from care and was included in all regression analyses as a time‐varying exposure. ART status was coded zero for never starting ART and one once ART has been started until the time of censoring. Interaction terms with time‐varying covariates were created for baseline variables that violated the proportional hazards assumption. Baseline covariates were excluded from the model when the inclusion of the interaction term did not resolve the proportional hazards assumption violation. The study protocol was reviewed and approved by the Institutional Review Boards of the University of Witwatersrand (M141103) and Boston University (H‐33516). Permission to perform the research was also obtained from the City of Johannesburg and the study clinics.

## 3 Results

### 3.1 Clinical and demographic characteristics at HIV diagnosis

We enrolled 425 of 580 screened participants into the study. Fifteen participants were excluded from the analytic dataset for being on ART before study enrolment (Figure 1). Overall, 144 (35.1%) participants were enrolled pre‐UTT, 178 (43.4%) during UTT and 88 (21.4%) under the SDI policy (Table 1). About half (56.1%) of participants were female with a median age of 33.5 (IQR: 28.3 to 39.4). While the sex difference favoured women in the pre‐UTT (59.0% vs. 41.0%) and UTT cohorts (57.9% vs. 42.1%), 47.7% of the SDI cohort is female and 52.3% male. About a quarter (27.8%) of participants had at least a grade 12 education and two‐thirds (66.3%) were in an unmarried relationship. Nearly half (55.6%) reported being unemployed, 84.6% had two or more adults in the home. The median CD4 count at HIV diagnosis was 237 cells/µL (IQR: 100 to 430), and 6.6% reported ever having tuberculosis.

**Table 1:** Demographic characteristics of HIV‐positive patients enrolled in the study by treatment guideline period at HIV diagnosis (n = 410)

	Pre‐UTT	UTT	SDI	Total
	n = 144 n (%)	n = 178 n (%)	n = 88 n (%)	N = 410 n (%)
Facility				
PHC 1	66 (45.8)	97 (54.5)	52 (59.1)	215 (52.4)
PHC 2	78 (54.2)	81 (45.5)	36 (40.9)	195 (47.6)
Sex				
Male	59 (41.0)	75 (42.1)	46 (52.3)	180 (43.9)
Female	85 (59.0)	103 (57.9)	42 (47.7)	230 (56.1)
Age on the day of testing				
Median (IQR)	32.8 (27.3 to 37.7)	33.4 (28.3 to 39.2)	34.6 (30.6 to 42.9)	33.5 (28.3 to 39.4)
18 to 24 years	14 (9.7)	10 (5.6)	3 (3.4)	27 (6.6)
25 to 29 years	37 (25.7)	52 (29.2)	18 (20.5)	107 (26.1)
30 to 34 years	40 (27.8)	39 (21.9)	24 (27.3)	103 (25.1)
35 to 39 years	28 (19.4)	40 (22.5)	16 (18.2)	84 (20.5)
40+ years	25 (17.4)	37 (20.8)	27 (30.7)	89 (21.7)
Highest level of education				
Less than Grade 12	95 (66.0)	135 (75.8)	66 (75.0)	296 (72.2)
Grade 12 of higher	49 (34.0)	43 (24.2)	22 (25.0)	114 (27.8)
Marital status				
Single/no partner	28 (19.4)	22 (12.4)	9 (10.2)	59 (14.4)
Unmarried relationship	90 (62.5)	125 (70.2)	57 (64.8)	272 (66.3)
Married	21 (14.6)	24 (13.5)	19 (21.6)	64 (15.6)
Divorced/widowed	5 (3.5)	7 (3.9)	3 (3.4)	15 (3.7)
Employment status				
Unemployed	75 (52.1)	98 (55.1)	55 (62.5)	228 (55.6)
Employed	69 (47.9)	80 (44.9)	33 (37.5)	182 (44.4)
Number of adults in the household				
Lives alone	28 (19.4)	22 (12.4)	13 (14.8)	63 (15.4)
Two adults at home	80 (55.6)	108 (61.0)	62 (70.5)	250 (61.1)
Three or more adults	36 (25.0)	47 (26.6)	13 (14.8)	96 (23.5)
Travel time to PHC				
≤15 minutes	90 (62.5)	114 (64.0)	52 (59.1)	256 (62.4)
16 to 30 minutes	44 (30.6)	51 (28.7)	29 (33.0)	124 (30.2)
More than 30 minutes	10 (6.9)	13 (7.3)	7 (8.0)	30 (7.3)
Viral load suppressed (VL < 400 copies/mL) at six months				
No	9 (6.3)	13 (7.3)	1 (1.1)	23 (5.6)
Yes	48 (33.3)	87 (48.9)	39 (44.3)	174 (42.4)
Missing VL data	87 (60.4)	78 (43.8)	48 (54.6)	213 (52.0)
Baseline CD4 count (cells/mm3)				
Median (IQR)	248 (103 to 423)	233 (92 to 446)	237 (132 to 415)	237 (100 to 430)
≤200	61 (42.4)	65 (36.5)	33 (37.5)	159 (38.8)
201 to 350	33 (22.9)	34 (19.1)	16 (18.2)	83 (20.2)
351 to 500	25 (17.4)	29 (16.3)	11 (12.5)	65 (15.9)
>500	19 (13.2)	26 (14.6)	13 (14.8)	58 (14.1)
Missing	6 (4.2)	24 (13.5)	15 (17.0)	45 (11.0)
Policy months at HIV diagnosis (months after the date of policy announcement)				
<3 month	0	0	43 (71.7)	43 (11.8)
3 to 6 months	24 (18.9)	0	17 (28.3)	41 (11.2)
>6 months	103 (81.1)	178 (100.0)	0	281 (77.0)
Time to ART start				
No ART	57 (39.6)	38 (21.4)	18 (20.5)	113 (27.6)
Same‐day ART	0	3 (1.7)	14 (15.9)	17 (4.1)
1 to 14 days ART	13 (9.0)	79 (44.4)	42 (47.7)	134 (32.7)
15 to 30 days ART	41 (28.5)	34 (19.1)	9 (10.2)	84 (20.5)
>30 days ART	33 (22.9)	24 (13.5)	5 (5.7)	62 (15.1)
TB diagnosis (ever)				
Yes	13 (9.0)	12 (6.8)	2 (2.3)	27 (6.6)
No	131 (91.0)	165 (93.2)	86 (97.7)	382 (93.4)

### 3.2 ART initiation 12 months after HIV diagnosis

About a quarter of participants (27.6%) did not initiate ART in the 12 months of observation. ART initiation increased over time from 69.8% (95% CI: 61.0 to 77.2) among ART eligible pre‐UTT participants (ART eligible at CD4 ≤ 500) to 80.5% (95% CI: 72.6 to 86.5) and 88.3% (95% CI: 77.1 to 94.6) among comparable CD4 ≤ 500 participants under the UTT and SDI policy periods respectively. Overall ART initiation proportions were 60.4% (95% CI: 52.1 to 68.2), 78.7% (95% CI: 72.0 to 84.1) and 79.5% (69.6%, 95% CI: 69.6 to 86.9) for the pre‐UTT, UTT and SDI periods respectively. In total, 235/297 (79.1%) of ART initiates started ART in the first 30 days after HIV diagnosis (62.1%, 82.9% and 92.9% in the pre‐UTT, UTT and SDI cohorts respectively).

About half of all participants were missing six months viral load (VL) testing data: 60.4%, 43.8% and 54.6% in the pre‐UTT, UTT and SDI cohorts respectively. Among those with six months VL data, 88.3% were virally suppressed, 83.9 (95% CI: 74.0 to 93.9) pre‐UTT, 84.9% (95% CI: 76.5 to 93.3) under UTT and 96.6% (95% CI: 89.5 to 103.6) under SDI.

### 3.3 Patient attrition 12 months after HIV diagnosis

Overall, 178 (43.4%) participants were considered LTFU at 12 months (Table 2). Among these, 163 (91.6%) were lost from the diagnosing site within six months after HIV diagnosis. Total attrition at 12 months decreased from 47.2% pre‐UTT (95% CI: 38.9 to 48.2) to 38.2% under UTT (95% CI: 31.0 to 45.5) and then increased to 47.7% under the SDI policy (95% CI: 37.1 to 58.4) (Table 2). Attrition in the ART eligible pre‐UTT group (CD4 ≤ 500) (49.6%, 95% CI: 40.5 to 58.7) ) was similar to the proportion among the same population under the UTT (46.9%, 95% CI: 38.3 to 55.6) and SDI policies (48.3%, 95% CI: 35.7 to 61.2).

**Table 2:** Demographic and clinical characteristics associated with becoming lost from care at 12 months post‐HIV diagnosis in a study of retention in care among HIV‐positive patients by treatment guideline period

	LTFU	Person‐years (PY)	Incidence per 100PY	Crude	Adjusted	Adjusted with ART status as a time‐varying exposure	
	n (%)		(95% CI)	HR (95% CI)	aHR (95% CI)	HR (95% CI)	aHR (95% CI)
Total	178 (43.4)	229.1	77.7 (67.1 to 90.0)				
ART guideline							
Pre‐UTT	68 (47.2)	72.5	93.8 (73.9 to 118.9)	1	1	1	1
UTT	68 (38.2)	109.6	62.0 (48.9 to 78.7)	0.7 (0.5 to 1.0)	0.7 (0.5 to 1.0)	1.1 (0.8 to 1.6)	1.2 (0.9 to 1.8)
SDI	42 (47.7)	47.0	89.3 (66.0 to 120.8)	0.9 (0.6 to 1.4)	1.0 (0.7 to 1.5)	1.6 (1.1 to 2.4)	1.9 (1.1 to 2.9)
Facility							
PHC 1	79 (36.7)	131.3	60.2 (48.3 to 75.0)	1	1	1	
PHC 2	99 (50.8)	97.9	101.2 (83.1 to 123.2)	1.5 (1.1 to 2.1)	1.6 (1.2 to 2.1)	1.2 (0.9 to 1.6)	
Sex							
Male	85 (47.2)	94.2	90.2 (72.9 to 111.6)	1		1	
Female	93 (40.4)	134.9	68.9 (56.3 to 84.5)	0.8 (0.6 to 1.1)		1.0 (0.7 to 1.3)	
Age on the day of testing							
18 to 24 years	12 (44.4)	15.0	79.8 (45.3 to 140.5)	0.8 (0.5 to 1.6)	0.7 (0.4 to 1.4)	0.7 (0.4 to 1.2)	0.7 (0.3 to 1.2)
25 to 29 years	56 (52.3)	53.3	105.1 (80.9 to 136.6)	1	1	1	1
30 to 34 years	51 (49.5)	52.0	98.1 (74.6 to 129.1)	0.9 (0.6 to 1.4)	0.9 (0.6 to 1.3)	0.9 (0.6 to 1.3)	0.8 (0.6 to 1.3)
35 to 39 years	31 (36.9)	51.3	60.4 (42.5 to 85.9)	0.7 (0.4 to 1.0)	0.7 (0.5 to 1.1)	0.7 (0.5 to 1.1)	0.6 (0.4 to 1.0)
40+ years	28 (31.5)	57.6	48.6 (33.6 to 70.4)	0.5 (0.3 to 0.8)	0.5 (0.3 to 0.8)	0.6 (0.4 to 1.0)	0.5 (0.3 to 0.8)
Highest level of education							
<Grade 12	122 (41.2)	172.1	70.9 (59.4 to 84.7)	1		1	
≥Grade 12	56 (49.1)	57.1	98.1 (75.5 to 127.5)	1.3 (0.9 to 1.7)		1.1 (0.8 to 1.5)	
Marital status							
Single/no partner	37 (62.7)	24.6	150.4 (109.0 to 207.5)	1	1	1	1
Unmarried relationship	111 (40.8)	156.6	70.9 (58.9 to 85.4)	0.6 (0.4 to 0.8)	0.5 (0.4 to 0.8)	0.6 (0.4 to 0.8)	0.5 (0.4 to 0.8)
Married	25 (39.1)	37.3	67.0 (45.3 to 99.1)	0.6 (0.3 to 0.9)	0.7 (0.4 to 1.1)	0.7 (0.4 to 1.2)	0.8 (0.5 to 1.3)
Divorced/widowed	5 (33.3)	10.6	47.0 (19.6 to 112.9)	0.4 (0.2 to 1.1)	0.4 (0.2 to 1.2)	0.6 (0.3 to 1.6)	0.8 (0.3 to 2.1)
Employment status							
Unemployed	93 (40.8)	130.3	71.4 (58.2 to 87.5)	1		1	
Employed	85 (46.7)	98.9	86.0 (69.5 to 106.4)	1.2 (0.9 to 1.6)		1.1 (0.8 to 1.4)	
Number of adults in the household							
Lives alone	28 (44.4)	34.4	81.4 (56.2 to 117.9)	1		1	
Two adults	107 (42.8)	139.9	76.5 (63.3 to 92.4)	1.0 (0.6 to 1.4)		1.0 (0.7 to 1.7)	
≥Three adults	43 (44.8)	53.9	79.8 (59.2 to 107.7)	1.0 (0.6 to 1.6)		1.0 (0.6 to 1.6)	
Travel time to PHC							
≤15 minutes	106 (41.4)	146.0	72.6 (60.0 to 87.8)	1		1	1
16 to 30 minutes	56 (45.2)	67.5	83 (63.9 to 107.9)	1.1 (0.8 to 1.6)		1.0 (0.7 to 1.5)	1.1 (0.8 to 1.5)
≥30 minutes	16 (53.3)	15.7	102.1 (62.6 to 166.7)	1.3 (0.8 to 2.2)		1.7 (1.0 to 1.6)	1.8 (1.1 to 3.1)
Baseline CD4 count (cells/mm^3^)							
≤200	61 (38.4)	95.6	63.8 (49.7 to 82.1)	1	1	1	1
201 to 350	42 (50.6)	43.4	96.7 (71.4 to 130.8)	1.4 (1.0 to 2.1)	1.4 (1.0 to 2.1)	1.2 (0.8 to 1.7)	1.2 (0.8 to 1.7)
351 to 500	24 (36.9)	40.9	58.7 (39.4 to 87.6)	0.9 (0.6 to 1.5)	1.0 (0.6 to 1.5)	0.9 (0.6 to 1.5)	0.8 (0.5 to 1.4)
>500	25 (43.1)	30.7	81.4 (55.0 to 120.5)	1.3 (0.8 to 2.0)	1.4 (0.9 to 2.3)	0.9 (0.6 to 1.4)	0.9 (0.6 to 1.5)
Missing	26 (57.8)	18.6	139.9 (95.3 to 205.5)	1.9 (1.2 to 3.0)	2.1 (1.3 to 3.5)	1.0 (0.6 to 1.6)	0.8 (0.5 to 1.3)
Policy‐months at HIV diagnosis (months after the date of policy announcement)							
<3 month	45 (51.1)	45.3	99.4 (74.2 to 133.1)	1		1	
3 to 6 months	19 (46.3)	20.9	91.1 (58.1 to 142.8)	0.9 (0.6 to 1.6)		0.7 (0.4 to 1.2)	
>6 months	114 (40.6)	163.0	69.9 (58.2 to 84.0)	0.8 (0.6 to 1.1)		0.7 (0.5 to 1.0)	
On‐ART at the end of follow‐up (time‐varying)^a^							
No	102 (90.3)	12.1	846.7 (697.4 to 1028.1)			1	1
Yes	76 (34.4)	217.1	35.0 (28.0 to 43.8)			0.1 (0.1 to 0.1)	0.5 (0.2 to 1.3)
TB diagnosis (ever)							
Yes	14 (51.9)	14.0	99.7 (59.0 to 168.3)	1		1	
No	163 (42.7)	215.0	75.8 (65.0 to 88.4)	0.8 (0.5 to 1.4)		0.8 (0.5 to 1.4)	

CD4, Cluster of differentiation four; LTFU, Lost to follow‐up; PHC, Primary Healthcare clinic; SDI, Same‐day Initiation; TB, Tuberculosis; UTT, Universal Test and Treat.

^a^ On‐ART at the end of follow‐up was the time‐varying ART status throughout the follow‐up period (changed from 0 to 1 upon ART initiation).

### 3.4 Risk and predictors of becoming LTFU 12 months after HIV diagnosis

Patient attrition was 30% lower in the UTT guideline period compared to the pre‐UTT period (aHR 0.7, 95% CI: 0.5 to 1.0) (Table 2). However, the total attrition was similar between the SDI and pre‐UTT cohorts (aHR 1.0, 95% CI: 0.7 to 1.5). There was a marked difference in attrition by diagnosing clinic, with participants from PHC2 being 60% more likely to becomes LTFU at 12 months (aHR 1.6, 95% CI: 1.2 to 2.1).

The risk of becoming LTFU at 12 months decreased with increasing age at HIV diagnosis, with participants older than 40 years old being nearly 50% less likely to become lost compared to those in the 25 to 29 years age group (aHR 0.5, 95% CI:0.3 to 0.8). Compared to participants reporting being single, those who were in a non‐marital relationship were 50% less likely to be lost from care (aHR 0.5, 95% CI: 0.3 to 0.8).

Patients who were missing CD4 count data were twice more likely to be lost at 12 months (aHR 2.1, 95% CI: 1.3 to 3.5). Also, patients who presented with CD4 count 201 to 350 cells/mm3 were more likely to be LTFU compared to those who had baseline ≤200 cells/mm [3], with no difference with higher CD4 categories. Having a history of tuberculosis was also not associated with attrition from care. However, participants who travelled for at least 30 minutes to the diagnosing clinic were more likely to be LTFU compared to those with ≤15 minutes of travel time (aHR 1.8, 95% CI:1.1 to 3.1).

### 3.5 Patient attrition before and after ART initiation

**Figure 2:**
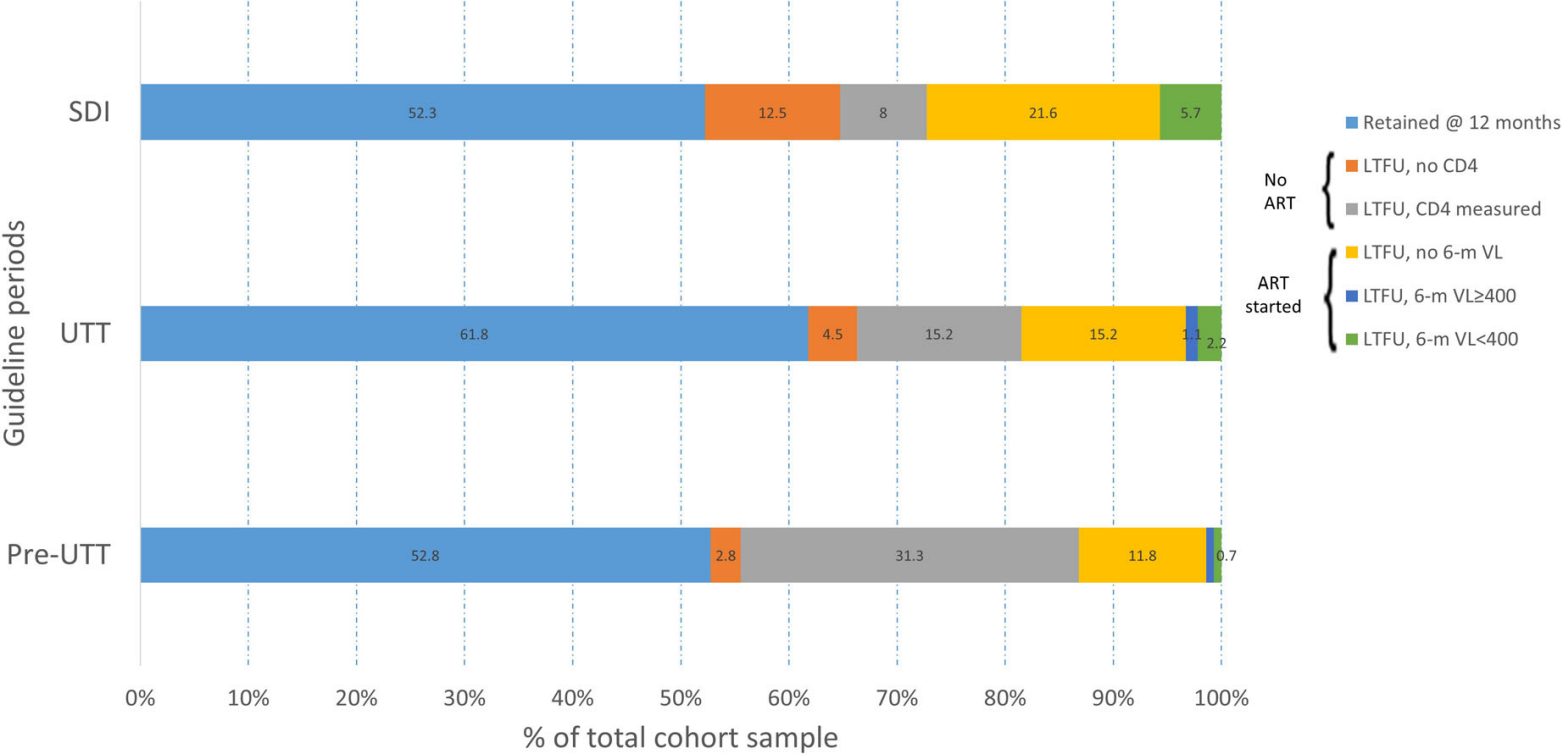
Participants retention and stages of the HIV care cascade when patients dropped from care in the first 12 months after HIV diagnosis.

Among participants who were LTFU at 12 months, 57.3% had not started ART, 37.1% started ART but had an unsuppressed/missing VL at six months. Only 5.6% had started ART and received a suppressed six‐month VL test result. Among participants who were LTFU at 12 months, the proportion of patients lost after ART initiation increased from 27.8.0% pre‐UTT to 48.4% in the UTT cohort and highest in the SDI cohort at 57.2% (Figure 2). The median time on ART at the last visit, among those lost after ART initiation, was 8.7 months (IQR: 5.7 to 10.0) pre‐UTT, 10.0 months (IQR: 6.9 to 11.0) under UTT and 8.7 months (IQR: 4.5 to 10.6) under the SDI policy.

In survival analyses adjusted for the time‐varying exposure to ART, compared to the pre‐ART phase of care, on‐ART attrition from care was similar among pre‐UTT and UTT participants (aHR 1.2, 95% CI 0.9 to 1.8) (Table 2). However, the on‐ART risk of LTFU was 90% higher among participants diagnosed under the SDI policy compared to pre‐UTT (aHR 1.9, 95% CI: 1.1 to 2.9).

## 4 Discussion

This is one of the first studies to look at attrition along the entirety of the HIV cascade, including before ART initiation, pre‐UTT, under UTT and SDI policies in South Africa. We aimed to determine whether the implementation of the UTT and SDI policies were associated with reduced 12‐month attrition from HIV care for newly diagnosed HIV‐positive patients in Johannesburg. Similar to other studies in sub‐Saharan Africa, we found a marked improvement in early treatment initiation rates after the implementation of the general UTT policy [12, 28, 29, 30]. We also found that total attrition remained substantial across policy periods, with levels around 40%. Total attrition declined after the introduction of the UTT policy but returned to pre‐UTT levels in the SDI period. The data also show a marked shift in the patterns of patient attrition from the pre‐ART period, mainly before UTT, to the on‐ART phase of patient care experience under the SDI policy.

The evidence for the impact of the SDI policy implementation in South Africa on patient attrition is mixed. Clinical trials, including the RapIT trial in South Africa, have demonstrated that same‐day ART improved viral suppression rates but showed limited evidence for improved retention in care [12, 22, 31, 32]. The recent SLATE trial in South Africa and Kenya also showed that a simple algorithm for same‐day treatment initiation could increase early ART uptake but not necessarily short term retention in care [12]. Still, in observational studies in South Africa, accelerated ART initiation resulted in a trend towards an increased risk of being lost to follow‐up at six months [7, 32].

The UTT policy effectively removed the CD4‐based eligibility criteria for ART without eliminating all ART preparation counselling sessions that were previously offered to clients. Yet, facilities began to shorten the time to ART initiation immediately after the introduction of UTT [28]. However, despite the hard push for SDI after September 2017, only 20% of participants started ART on the day of diagnosis, with the majority starting within a week [28]. Furthermore, the time pressure introduced by the SDI policy possibly resulted in the elimination and overall decline in the quality of psychosocial support offered to HIV positive clients [33, 34, 35]. A qualitative study with HIV positive patients retained in HIV care for over ten years showed that the clinic environment, support networks and self‐efficacy for treatment adherence were critical facilitators of staying in care [33, 34, 35, 36]. In an observational cohort study, enrolling patients diagnosed with HIV under the SDI policy, we demonstrated that social preparation before HIV testing (disclosure of intention to test, partner/family support at the clinic on the day of HIV testing) were essential predictors of ART uptake, particularly among men [37]. The effect of social preparation for ART after HIV testing on long‐term retention in care needs further investigation. Nevertheless, improved patient education with regards to the benefits of early ART initiation and consistent adherence support is essential to increase patient retention under the SDI policy. Additional support is most pertinent in the first six months of care, as our data and current evidence indicate that patient losses are more substantial in the first six months of the care experience [12].

Our findings showed that older age and being in a non‐marital relationship were associated with lower attrition rates, suggesting the need for support in managing HIV treatment for those who may have to disclose to a partner. Interestingly, similar to the RapIT study results, a higher proportion of men was diagnosed with HIV and initiated ART, suggesting that the offer of immediate ART may be more attractive to men than women [14]. Being in a partnership may signify access to social support in some cases, but being in an insecure partnership may amplify fears associated with HIV status disclosure, and limit ART adherence [38, 39, 40, 41, 42, 43, 44]. Fears of HIV stigma also compel some patients to access HIV care further away to avoid being identified at a nearby clinic [45]. As expected, our study found that travelling for at least 30 minutes to the clinic was associated with higher attrition, highlighting the challenge that longer travel‐time poses for accessing care consistently [26, 46].

Although the study sites were located within the same community and served the same population, there was a marked difference in mostly pre‐ART attrition between the two study site. These differences highlight the potential variability across facilities, depending on internal site processes and patients’ rapport with clinic staff, indicating the need for training and standardization of services offerings across clinics. We reported similar variations with regards to the implementation of SDI in the Gauteng province of South Africa [28]. Overall, attrition analyses were adjusted for facility differences which would have addressed the facility‐specific contextual changes. There was no facility‐specific difference in on‐ART patient attrition.

There are several limitations to this study. First, this was not a randomized trial, but we compared outcomes across ART guidelines policies periods in South Africa. The study periods do not define exact interventions that affected patient attrition but provide a general assessment of HIV care practices that were largely guided by the ART policy. Second, we only included patients who were diagnosed at two primary care clinics through facility‐based testing, indicating their motivation to obtain health services. These participants may perceive a higher need or fewer barriers for accessing HIV care than those who did not seek care. Thus, our results do not reflect retention outcomes of the South African HIV‐infected population as a whole as system‐wide retention estimates that take into account clinic changes may be higher. Thirdly, the change in the interview language options resulted in fewer exclusions in the UTT and same‐day ART cohorts. However, there was no difference in the demographic characteristics such as highest education and employment levels, indicating that the populations were broadly comparable on most baseline factors. Finally, the sample size for each cohort was based on assumptions about potential improvements in patient retention, and a larger sample size would have provided more precise and robust retention estimates. Nevertheless, we report considerable attrition from care under the SDI policy, indicating that expanding access to ART is not sufficient to ensure long‐term retention in care.

## 5 Conclusions

Although attrition from care decreased slightly following the introduction of UTT, the potential increases in on‐ART patient attrition after the introduction of the SDI policy is cause for concern. Overall, two‐fifths of newly diagnosed HIV patients are lost to care within 12‐month post‐HIV diagnosis under the new SDI policy. These findings add to the growing body of evidence that highlights the urgent need for research to determine the best way for rapidly initiating patients on ART without compromising long‐term retention in HIV care.

## Competing Interests

The authors have declared that no competing interests exist.

## Authors’ Contributions

DO and MPF conceptualized the study and paper. CH and TS managed the study implementation and conducted the primary data analysis and contributed to the result interpretation. LL, JB and MM contributed to the interpretation of the results. All authors reviewed and approved the final manuscript.
